# l-Glutamine and Whole Protein Restore First-Phase Insulin Response and Increase Glucagon-Like Peptide-1 in Type 2 Diabetes Patients

**DOI:** 10.3390/nu7042101

**Published:** 2015-03-24

**Authors:** Dorit Samocha-Bonet, Don J. Chisholm, Jens J. Holst, Jerry R. Greenfield

**Affiliations:** 1 Diabetes & Metabolism Division, Garvan Institute of Medical Research, 384 Victoria Street, Darlinghurst 2010, Sydney, NSW, Australia; E-Mails: d.chisholm@garvan.org.au (D.J.C.); j.greenfield@garvan.org.au (J.R.G.); 2 Faculty of Medicine, UNSW Australia, High Street, Kensington 2052, Sydney, NSW, Australia; 3 NNF Center for Basic Metabolic Research, Department of Biomedical Sciences, University of Copenhagen, Panum Institute, Nørregade 10, DK-1165 Copenhagen, Denmark; E-Mail: jjholst@sund.ku.dk; 4 Department of Endocrinology and Diabetes Center, St. Vincent’s Hospital, 390 Victoria Street, Darlinghurst 2010, Sydney, NSW, Australia

**Keywords:** glutamine, insulin response, hyperglycemic glucose clamp, type 2 diabetes

## Abstract

l-glutamine triggers glucagon-like peptide-1 (GLP-1) release from L cells *in vitro* and when ingested pre-meal, decreases postprandial glycaemia and increases circulating insulin and GLP-1 in type 2 diabetes (T2D) patients. We aimed to evaluate the effect of oral l-glutamine, compared with whole protein low in glutamine, on insulin response in well-controlled T2D patients. In a randomized study with a crossover design, T2D patients (*n* = 10, 6 men) aged 65.1 ± 5.8, with glycosylated hemoglobin (HbA1c) 6.6% ± 0.7% (48 ± 8 mmol/mol), received oral l-glutamine (25 g), protein (25 g) or water, followed by an intravenous glucose bolus (0.3 g/kg) and hyperglycemic glucose clamp for 2 h. Blood was frequently collected for analyses of glucose, serum insulin and plasma total and active GLP-1 and area under the curve of glucose, insulin, total and active GLP-1 excursions calculated. Treatments were tested 1–2 weeks apart. Both l-glutamine and protein increased first-phase insulin response (*p* ≤ 0.02). Protein (*p* = 0.05), but not l-glutamine (*p* = 0.2), increased second-phase insulin response. Total GLP-1 was increased by both l-glutamine and protein (*p* ≤ 0.02). We conclude that oral l-glutamine and whole protein are similarly effective in restoring first-phase insulin response in T2D patients. Larger studies are required to further investigate the utility of similar approaches in improving insulin response in diabetes.

## 1. Introduction

Glucagon like peptide-1 (GLP-1) secreted from gastrointestinal L cells has a major role in mediating physiological insulin release following a meal [1,2]. Numerous clinical studies suggest that well-controlled type 2 diabetes patients exhibit an intact GLP-1 secretion pattern, comparable to that of healthy individuals [3]. Furthermore, insulin release from the beta cell in response to endogenous GLP-1 is preserved in well-controlled type 2 diabetes [4]. Therefore, there has been much recent interest in developing methods by which GLP-1 action can be enhanced in diabetes. Glutamine is the most efficacious amino acid to trigger GLP-1 release from the L cells model GLUTag cells [5] and native murine L cells *ex vivo* [6]. In type 2 diabetes patients, ingestion of l-glutamine prior to a meal increases circulating GLP-1 [7,8,9], delays gastric emptying [8], increases circulating insulin and lowers postprandial glycaemia [9]. While glutamine is the most efficacious amino acid to induce GLP-1 release from L cells, most amino acids exhibit insulinotropic effects [10].

The aim of the present study was to compare the effects of oral glutamine with whole protein low in glutamine on: (i) first- and second- phase insulin response using the gold standard hyperglycemic glucose clamp; and (ii) total and active GLP-1 in well-controlled type 2 diabetes patients. We hypothesized that glutamine would be more efficacious than whole protein in restoring insulin response due to greater increases in GLP-1.

## 2. Experimental Section

### 2.1. Participants

Type 2 diabetes patients were recruited through advertisements at the St Vincent’s Hospital precinct, Sydney and in local newspapers. Inclusion criteria were age 40–70 years, diabetes duration of 5 years or less, treatment with diet or metformin in a stable dose (≤2000 mg/day, for at least 3 months), glycosylated hemoglobin (HbA_1c_) 6.5%–9% (48–75 mmol/mol), body mass index (BMI) 40 kg/m^2^ or less and stable body weight in the preceding 6 months (±2 kg). Exclusion criteria were treatment with oral hypoglycemic agents other than metformin, ethanol intake >20 or 40 g/day for women and men, respectively, liver or kidney disease or abnormal full blood count, renal or liver function tests, use of weight loss medications, previous bowel surgery or documented malabsorption. The study was approved by the Human Research and Ethics Committee at St Vincent’s Hospital and participants gave written informed consent prior to commencement of the study. The study was registered at ClinicalTrials.gov (NCT-00673894).

### 2.2. Study Design

The effect of glutamine compared with protein and water were evaluated in a randomized crossover study. Participants attended the Clinical Research Facility after an overnight fast on three separate occasions 1–2 weeks apart in a random order. The effects of: (1) 25 g l-glutamine (Cambridge Commodities, Cambridge, UK) supplemented with 16 g Philadelphia cheese (26 g protein, 5 g fat, 152 kcal; Gln treatment); or (2) 200 g low fat cottage cheese (25 g protein, 2 g Gln [11], 5 g fat and 182 kcal; Protein treatment); or (3) water on the study endpoints were investigated.

### 2.3. Study Procedures

Two intravenous cannulas were inserted for glucose infusion and blood withdrawal. Glutamine was mixed in cold water (250 mL) and the same water volume was given in all studies. Blood was drawn fasting twice 10 min apart (averaged concentrations are presented for all endpoints), then treatment ingested within 8 min and blood collected at t = 15 and 30 min. At t = 30, glucose (0.3 g/kg, maximum 25 g; 25% glucose, Baxter Healthcare, Old Toongabbie, NSW, Australia) was injected intravenously over 1 min followed by frequent blood sampling for 10 min (t = 30–40). Hyperglycemia was then maintained (target 10.8 mmol/L) for additional 110 min (t = 40–150 min) by adjusting glucose infusion rate (GIR) according to 10 min blood glucose readings.

### 2.4. Laboratory Analyses

Blood glucose (Yellow Springs Instrument Company; Life Sciences) and serum insulin (radioimmunoassay; Millipore, St Charles, IL, USA) were measured at t = 0, 15 and 30 min post Gln/protein/water treatment, at t = 1, 2, 3, 4, 5, 6, 8 and 10 min post intravenous glucose bolus (t = 30–40 min) and at t = 60, 90, 120, 130, 140 and 150 min during the hyperglycemic glucose clamp. Blood samples for total and active GLP-1 were collected into EDTA-coated tubes, with dipeptidyl peptidase (DPP)4 inhibitor and trasylol in the active GLP-1 testing tube, and immediately centrifuged for 7 min at 4100 *g*, snap frozen, and stored at −80 °C until analysis. Total GLP-1 was measured by radioimmunoassay after extraction of plasma with 70% ethanol. Carboxy-terminal GLP-1 immunoreactivity was determined using antiserum 89390. Active GLP-1 was analyzed using an ELISA, as previously described [12]. Total and active GLP-1 plasma concentrations were measured at t = 0, 15 and 30 min post Gln/protein/water treatment and at t = 60, 90, 140 and 150 min during the hyperglycemic glucose clamp.

### 2.5. Statistical Analysis

Area under the curves (AUCs) of glucose, insulin and total and active GLP-1 excursions were calculated (baseline concentrations of these endpoints were not significantly different between treatments, *p* > 0.1). AUCs were calculated for the 30 min after ingestion of Gln/protein/water (AUC_0–30 min_), 10 min post IV glucose injection (t = 30–40; AUC_IVGTT_; first-phase insulin response) and for the hyperglycemic glucose clamp (t = 40–150 min; AUC_Clamp_; second-phase insulin response). Data are presented as means ± SD, unless stated otherwise. Insulin data were log_10_-transformed prior to statistical analysis. One-way ANOVA with Tukey posthoc comparisons was used to test for differences in AUCs of endpoints between l-glutamine, whole protein and water. Data were analyzed using SPSS version 21 (IBM Corp., Armonk, NY, USA).

## 3. Results

Well-controlled type 2 diabetes patients (*n* = 10, 6 men, 4 women, all postmenopausal) participated in the study. Participants were 65.1 ± 5.8 years old with diabetes duration of 3.5 ± 1.5 years, BMI 27.1 ± 2.4 kg/m^2^ and HbA1c 6.6% ± 0.7% (48 ± 8 mmol/mol).

### 3.1. Blood Glucose Response

**Figure 1:**
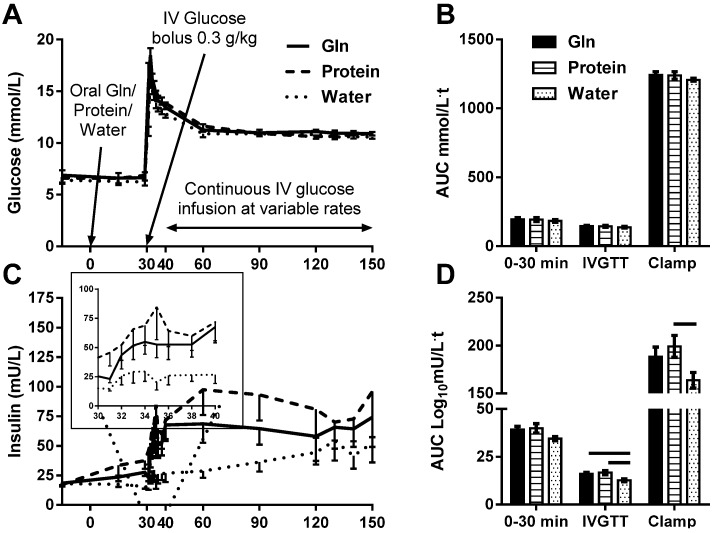

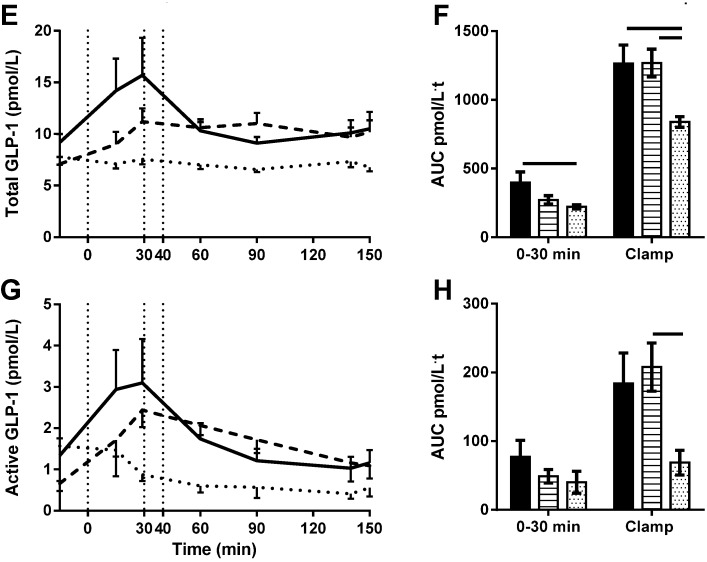
The effect of l-glutamine (Gln), protein or water on blood glucose (**A** and **B**), serum insulin (**C**; first-phase insulin response, inset, and **D**) and plasma total (**E** and **F**) and active (**G** and **H**) GLP-1 excursions and area under the curve (AUC), respectively in response to intravenous glucose bolus and hyperglycemic glucose clamp. AUC of the response of glucose, insulin and total and active GLP-1 at t = 0–30 min (30 min post treatment ingestion), t = 30–40 min (10 min post glucose injection) and t = 40–150 min (during hyperglycemic glucose clamp) were calculated and differences between treatments tested by one-way ANOVA with Tukey posthoc analyses. Data are mean ± SEM. Horizontal lines above AUC bars indicate statistical significance (*p* ≤ 0.05).

The treatments were well tolerated. Baseline blood glucose was 6.6 ± 1.3 mmol/L, without significant differences between treatments (*p* = 0.7). Blood glucose peaked 2 min after the IV glucose bolus (17.3 ± 2.7 mmol/L), without significant differences between treatments (*p* = 0.2). Blood glucose was clamped at an average of 10.8 ± 0.4 mmol/L during clamp steady state (t = 120–150 min), without a significant difference between treatments (*p* = 0.3). Glucose AUCs were not significantly different between the treatments (*p* ≥ 0.5, Figure 1A,B). Glucose infusion rate (GIR) and GIR normalized to body weight necessary to maintain hyperglycemia were not significantly different between treatments (*p* > 0.25), consistent with similar acute effects of the treatments on insulin sensitivity.

### 3.2. Serum Insulin Response

Insulin AUC_0-30min_ was not significantly different between treatments (*p* = 0.1; Figure 1C,D). First-phase insulin response (Insulin AUC_IVGTT_) was blunted after water and augmented by both Gln (*p* = 0.02) and protein (*p* = 0.01; Figure 1C inset and D). Second-phase insulin response (Insulin AUC_Clamp_) was significantly augmented by protein (*p* = 0.05), but not Gln (*p* = 0.2) compared with water (Figure 1C,D).

### 3.3. Plasma Glucagon-Like Peptide 1 Response

Total GLP-1 AUC_0-30min_ was augmented by Gln (*p* = 0.05), but not protein (*p* = 0.8) compared with water. During the hyperglycemic glucose clamp, total GLP-1 remained significantly increased after both Gln (*p* = 0.02) and protein (*p* = 0.02) compared with water (Figure 1E,F). Active GLP-1 AUC_0–30min_ was not significantly different between treatments (*p* = 0.3; Figure 1G,H). During the hyperglycemic clamp, active GLP-1 was increased after protein (*p* = 0.03) and tended to increase after Gln (*p* = 0.08) compared with water (Figure 1G,H).

## 4. Discussion

We demonstrated that glutamine and whole protein low in glutamine ingested prior to intravenous hyperglycemia were similarly effective in restoring first-phase insulin response in a cohort of well-controlled type 2 diabetes patients. First- and second- phase insulin secretion are impaired in type 2 diabetes and under hyperglycemic glucose clamp conditions, endogenous GLP-1 is reported to augment both phases of insulin secretion in response to duodenal nutrition perfusion [13]. van der Klaauw and colleagues have recently reported that high protein mixed meals were more efficacious in enhancing GLP-1 excursions postprandially compared with isocaloric high fat or high carbohydrate mixed meals in healthy volunteers, an effect potentially attributed to glutamine [14]. No previous study however, directly compared the effect of isocaloric whole protein and l-glutamine on GLP-1 response.

We report that glutamine was more efficacious in increasing GLP-1 in the circulation 30 min post ingestion, but that both glutamine and whole protein low in glutamine restored first-phase insulin response. Other incretins were not measured in the present study and could have explained the augmented insulin response associated with the protein ingestion. In particular, gastric inhibitory polypeptide (GIP) could have been enhanced by ingestion of whole protein [15]. While previous studies suggested impaired insulinotropic effect of GIP in type 2 diabetes patients [16], a recent study proposed a certain degree of contribution of GIP to the incretin-stimulated insulin secretion observed in type 2 diabetes patients using the hyperglycemic glucose clamp together with duodenal nutrition perfusion [13]. Another potential contributor to the augmented insulin response in the whole protein treatment in the present study is whey protein [17,18]. The augmented second-phase insulin response observed with whole protein ingestion in the present study is consistent with the increase in intact GLP-1 in the circulation during this time.

Previously, we have reported that a similar dose of glutamine ingested with a meal increased the early active GLP-1 response, but not the early insulin response to the meal [9]. However in the present study, glutamine increased circulating GLP-1 and restored first-phase insulin response to an IV glucose challenge. Differences between these finding are likely attributed to the different stimuli, namely a mixed meal administered orally *versus* glucose administered IV. Furthermore, increases of approximately 1.5-fold in circulating GLP-1 concentrations in the present study were related to first-phase insulin restoration. In support, similar scale increases in circulating GLP-1 in response to OGTT were associated with restoration of first-phase insulin response to IVGTT in severely obese type 2 diabetes patients undergoing bariatric surgery [19].

The main strengths of this study are the use of the hyperglycemic glucose clamp, enabling the investigation of the treatments on first- and second- phase insulin response. Furthermore, the test treatments were energy and fat matched to negate possible effects of energy and fat on gastric emptying, insulin and incretin response. Furthermore, the randomized crossover design increased the power to detect differences in response in a relatively small cohort. The two main limitations were the lack of a healthy control group and the relatively small cohort studied.

## 5. Conclusions

Both l-glutamine and whole protein restored first-phase insulin response in type 2 diabetes patients. Larger studies are required to further investigate the utility of similar approaches in improving insulin secretory capacity in type 2 diabetes.
